# The Development and Evaluation of the Interprofessional Education Facilitation Program for Health Professionals Using the Attention, Relevance, Confidence, and Satisfaction (ARCS) Model of Instructional Design

**DOI:** 10.7759/cureus.37496

**Published:** 2023-04-12

**Authors:** Daisuke Son, Kazumi Kawamura, Miho Utsumi, Mitsuko Nakashima, Kanako Suzuki, Naho Watanabe

**Affiliations:** 1 Department of Community-Based Family Medicine, Faculty of Medicine, Tottori University, Yonago, JPN; 2 Administration Division, iDesignLabo, Toyohashi, JPN; 3 Faculty of Pharmaceutical Sciences, Kobe Gakuin University, Kobe, JPN; 4 Higashigaoka Faculty of Nursing, Tokyo Healthcare University, Tokyo, JPN; 5 Administration Division, 4UrSMILE, Tokyo, JPN; 6 Faculty of Nursing, Toho University, Tokyo, JPN

**Keywords:** program development, arcs model, instructional design, interprofessional education facilitation, interprofessional education and collaboration

## Abstract

Background

It is important to learn interprofessional education (IPE) facilitation skills to promote interprofessional collaboration in healthcare. Nonetheless, to date, only a handful of IPE facilitation programs have been developed through research.

Objective

The objective of this study was to create an IPE facilitation program for healthcare professionals who wanted to promote interprofessional collaboration in their organizations based on the tenets of instructional design and evaluate its effectiveness.

Methods

This study’s methodology was a mixed method based on relative subjectivism. We developed a two-day IPE facilitation program to learn IPE facilitation skills and promote interprofessional collaboration in the participants’ own organizations. The program was developed based on the instructional design principles of the attention, relevance, confidence, and satisfaction (ARCS) model, measuring the participants’ Interprofessional Facilitation Scale (IPFS) scores at three time points: before the first day, after the second day, and approximately one year after the course was completed. A one-way analysis of variance test was used to compare IPFS means at the three time points, and open-ended statements were qualitatively analyzed using thematic analysis.

Results

Twelve healthcare providers (four physicians, two pharmacists, one nurse, one rehabilitation worker, one medical social worker, one clinical psychologist, one medical secretary, and one other) participated in the completed IPE facilitation program. Their IPFS scores increased significantly from 17.4 ± 16.1 before the program to 38.1 ± 9.4 after the program and remained at 35.1 ± 11.7 for one year (p* *= 0.008). In addition, qualitative analysis suggested that the knowledge and skills learned in the program could be applied in the participants’ workplaces, which helped them maintain their IPE facilitation skills.

Conclusion

We developed a two-day IPE facilitation program based on the ARCS instructional design model, and the participants’ IPE facilitation skills scores increased and were maintained one year later.

## Introduction

Interprofessional collaboration among healthcare professionals is of the utmost importance, as it fosters team-based work, which results in superior communication and coordination of care [1,2]. This approach can lead to enhanced patient outcomes and satisfaction [3-5] while also providing an avenue for mutual learning and skill sharing, which results in more effective treatment plans [2]. Additionally, interprofessional collaboration promotes safety and reduces errors by comprehensively considering all aspects of patient care [6]. In essence, interprofessional collaboration is pivotal to providing patient-centered, high-quality care in healthcare settings.

Interprofessional education (IPE) plays a crucial role in fostering collaboration among all professionals involved in patient care, ultimately leading to healthcare providers who are optimally prepared and primed for interprofessional teamwork [5]. Nonetheless, the implementation of IPE is fraught with challenges. One of the primary difficulties is a lack of familiarity with the professional competence of other professions, making it challenging for healthcare professionals to learn with and from one another [7]. Another challenge is to develop and implement inclusive IPE activities that are achievable within formal classrooms and informal clinical settings [8]. The facilitation of interprofessional student groups is both rewarding and challenging, as diverse groups of students rely on the facilitator for guidance [8]. It is often difficult to get out of the discipline-specific mindset and consider the perspectives of all health professionals when providing guidance. The success of IPE also depends on the facilitation skills of the educators. The Interprofessional Facilitation Scale (IPFS) is a tool to evaluate the facilitation skills of IPE educators [9]. The competence and confidence of IPE facilitators are vital for the success of IPE programs; thus, it is essential to understand their perspectives and explore their knowledge, skills, and attitudes toward effective IPE delivery [10]. IPE facilitators must be well-trained and possess the necessary knowledge, skills, and attitudes for effective IPE delivery [10] to produce a skilled and collaborative workforce that can meet the complex needs of healthcare practices and society. Planning, designing, and facilitating interprofessional learning can be challenging but achievable by creating authentic IPE activities. Effective facilitation requires a shared understanding of disciplinary knowledge related to student learning outcomes and a focus on interprofessional collaborative outcomes. Demonstrating appreciation and respect for the roles of other professions is also essential for successful IPE facilitation [8].

However, there is a lack of comprehensive evidence regarding IPE facilitation. Although there is literature analyzing the IPE facilitation process through qualitative research [11], there are no IPE facilitation programs developed through research to date. This study aimed to develop an IPE facilitation program for healthcare professionals to learn IPE facilitation skills, promote interprofessional collaboration in the participants’ own organizations through research, and determine whether the facilitation skills of participating professionals could be enhanced and sustained.

## Materials and methods

This study’s methodology was a mixed method based on relative subjectivism. We employed a mixed method approach in order to quantitatively measure the learning effects of the developed program on the participants and to explain the reasons for the changes through qualitative analysis.

Program development through instructional design

**Figure 1:**
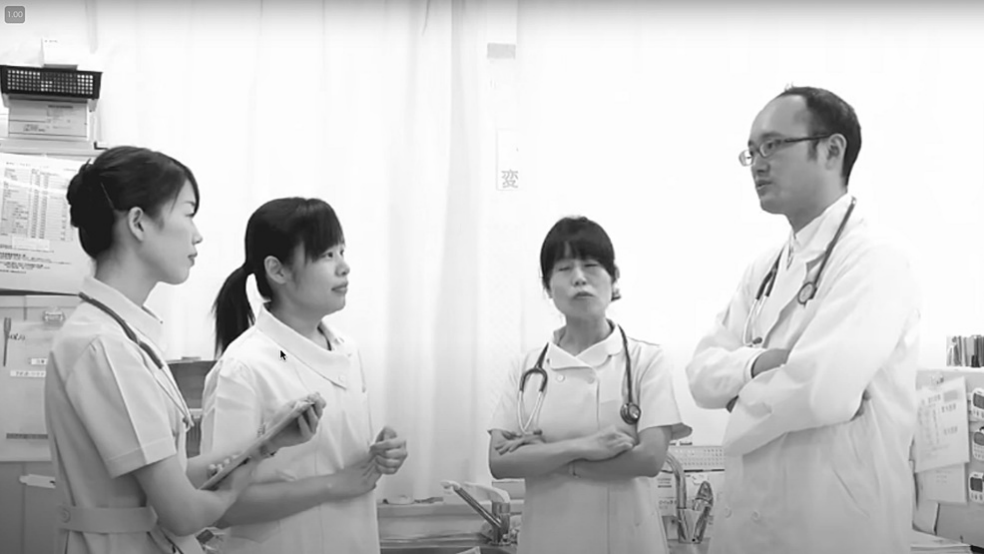
A scene from a video clip of a failed interprofessional collaboration.

We attempted to develop an IPE facilitation program based on the instructional design model. We designed a two-day IPE facilitation program that featured targeted footage of flawed interprofessional collaborations (Figure 1) as a viable approach for acquiring competence in IPE facilitation, which comprises advanced intellectual skills and attitudinal domains. The participants engaged in collective deliberation regarding the featured cases and contemplated how they could integrate the newly gained insights into their respective workplaces.

The attention, relevance, confidence, and satisfaction (ARCS) model has been validated as effective in enhancing learner motivation and learning outcomes in numerous studies; thus, we adopted the ARCS model of instructional design to develop our IPE facilitation program [12,13]. ARCS, a motivational model developed by John Keller in 1987, focuses on four categories of motivation: attention, relevance, confidence, and satisfaction [14]. Motivational concepts and characteristics are synthesized into four categories, forming the first significant part of the ARCS model. They also provide the basis for the second major feature of the ARCS model, the systematic design process comprising four steps: define, design, develop, and evaluate [13,14].

We designed the IPE facilitation program content to address the ARCS components.

Attention

This step involves capturing the learners’ attention and getting them interested in the course material. We generated interest from the participants by showing examples of failed interprofessional collaboration in video clips.

Relevance

This step focuses on demonstrating the significance of the material to the learners’ goals and interests. Our program targets professionals who want to become IPE facilitators in their organizations and enhance the interprofessional collaboration at their workplaces. The learning objectives are as follows: (1) to understand IPE facilitation skills and the barriers to interprofessional collaboration, (2) to analyze the barriers to interprofessional collaboration in the participants’ organizations, and (3) to design and facilitate an IPE program that can be applied in their organizations.

Confidence

This step aims to build the learners’ confidence in their ability to learn and apply the material. Our program provided opportunities to practice facilitation through mock workshops and tutor feedback on IPE facilitation.

Satisfaction

The final step entails providing opportunities for the participants to apply what they learned in the course and to receive feedback that increases their satisfaction with the learning experience. Our program was designed to help participants reflect on the challenges of interprofessional collaboration in their organizations and be able to apply the skills they learned in those contexts effectively.

**Figure 2:**
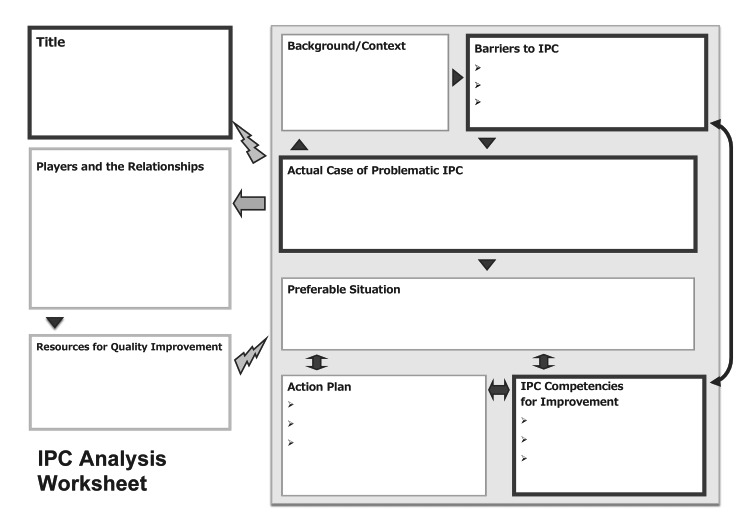
Interprofessional collaboration (IPC) analysis worksheet.

The pilot version of the two-day IPE facilitation program took place as follows: the first day consisted of (1) viewing and discussing a video of a case in which interprofessional collaboration did not go well, (2) a lecture on IPE facilitation skills and barriers to interprofessional collaboration, and (3) analyzing interprofessional collaboration in one’s organization using the interprofessional collaboration analysis worksheet (Figure 2). Then, the participants were assigned the task of designing a feasible IPE program for their organizations by the next day’s program, which would take place in 3-6 weeks. Whether the participants had implemented the program in their own organizations was confirmed in a post-program questionnaire after the second day of the program.

The second day consisted of (1) a discussion of the educational program designed by each participant, (2) a mock program to practice IPE facilitation, and (3) a feedback from the tutor on facilitation skills.

The evaluation of the participants’ IPE facilitation skills was conducted primarily as a formative assessment, with the tutors providing feedback on the participants’ performance during the mock workshop.

Participants and data collection

The target participants of the program were healthcare professionals who wanted to promote interprofessional collaboration and learn IPE facilitation skills in their organizations. They were invited to contact acquaintances of the first author (DS) through Facebook and other social networking sites. As a pilot program (beta test), 21 professionals (12 physicians, five nurses, two pharmacists, and two others) participated for two days in July and September 2014. After implementing the pilot version of the program, the participants identified areas for improvement. The participating health professionals provided input on the program’s content in a post-program questionnaire on feasibility and implementation.

To implement the final version of the IPE facilitation program, which was completed after content refinement, announcements were posted on social media sites such as Facebook and the mailing lists of related academic societies, and participants were recruited.

The IPE facilitation program was conducted with the participants who applied, and their facilitation skills were evaluated. The participants were asked to complete an online questionnaire: T1, before the day 1 program; T2, after the day 2 program three weeks later, and T3, approximately one year later; they self-assessed using the Japanese version of the Interprofessional Facilitations Scale (IPFS) [15], because the program was held in Japan and all the participants were Japanese. In the T3 questionnaire, they were asked to respond in free text to the following questions: What IPE facilitation did you practice during the first year after your participation? How did the program help you implement IPE facilitation? What were some of the difficulties you encountered in practicing IPE facilitation?

Data analysis

A nonparametric analysis, the Kruskal-Wallis one-way analysis of variance test, was performed to detect statistical differences in the means of the IPFS scores at the three time points. A Bonferroni analysis was performed as a post hoc analysis to identify significant differences between pairs at each time point. The significance level was set at 0.05. The Statistical Package for Social Sciences (SPSS) version 28 (IBM SPSS Statistics, Armonk, NY) was the statistical software used. The T3 questionnaire asked the participants to respond in an open-ended format about the IPE facilitation activities they have conducted in their own organizations in the past year, the lessons they have learned in their practice, and the barriers to their practice. Free-text answers were analyzed qualitatively using thematic analysis [16]. The thematic analysis approach follows a six-step process: familiarizing yourself with the data, generating initial codes, searching for themes, reviewing themes, defining and naming themes, and producing the report/manuscript [16]. Data analysis was performed mainly by the first author (DS) and triangulated with the other authors.

Ethical considerations

The participation in the program in this study was limited to those who were well-informed about the study’s purpose, significance, and protocol. The participants were duly informed of the protection of personal information. All participants understood and agreed to participate, and written informed consent was obtained from all the participants. This study was conducted with the approval of the Ethics Committee of the Japanese Primary Care Association (approval number H24-4). All participants consented to the publication of the results, as shown in Figure 1.

## Results

The final version of the IPE facilitation program was conducted in January and February 2015, three weeks apart. Twelve healthcare professionals (four physicians, two pharmacists, one nurse, one rehabilitation worker, one medical social worker, one clinical psychologist, one medical secretary, and one other) participated in the program. The participants were encouraged to use the worksheet to analyze the interprofessional collaboration challenges in their organizations during the three-week interval between days 1 and 2 and to try to implement IPE facilitation skills.

**Figure 3:**
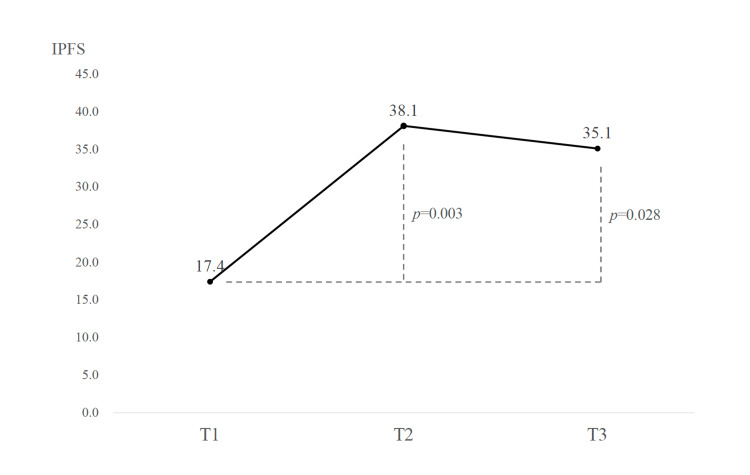
IPFS scores before and after program participation and one year later. IPFS: Interprofessional Facilitation Scale

The IPFS scores of the participants increased significantly after attending the program and were largely maintained after one year (Table 1 and Figure 3). Ten participants responded to the follow-up questionnaire administered one year after the program took place (there were two dropouts).

**Table 1:** Means and standard deviations (SD) of scores of IPFS at time points T1, T2, and T3. IPFS: Interprofessional Facilitation Scale

	N	Mean	SD
T1 (before day 1)	12	17.4	16.1
T2 (after day 2)	12	38.1	9.4
T3 (one year later)	10	35.1	11.7

The results of the Kruskal-Wallis test for the difference in the means of the IPFS at the three time points showed a significant difference (p = 0.008), and a post hoc analysis using the Bonferroni method showed substantial differences from T1 to T2 and from T1 to T3, with both effect sizes being greater than 1.0 (Table 2).

**Table 2:** Post hoc analysis of the difference in mean IPFS scores for each time pair and effect size. As a post hoc analysis, the Kruskal-Wallis test and the Bonferroni method were used. Effect size = Cohen’s d. IPFS: Interprofessional Facilitation Scale

Time pair	Standardized test statistics	Significance probability	Effect size
T2-T1	2.2	0.028	1.6
T3-T1	3.0	0.003	1.3
T3-T2	-0.6	0.51	0.28

The thematic analysis of the open-ended statements in the questionnaire applied one year after participation in the program revealed that the participants could apply what they learned to their workplaces (Table 3). The results suggest that the program improved their ability to analyze problems logically and their IPE facilitation skills, which contributed to conflict resolution between professionals, listening to and respecting other professionals, and eliciting initiatives from and motivating staff members. Conversely, the participants stated that barriers to IPE facilitation included disinterest of staff, power relations between professionals, established hierarchies based on years of experience, differences in perspectives between professions, and difficulties in maintaining IPE activities.

**Table 3:** Thematic analysis of open-ended statements in the questionnaire one year after program participation. IPE: interprofessional education

Learning in the program and application to the field
Ability to analyze problems logically
Improvement of IPE facilitation skills
Conflict management skills
Listening to other professionals
Eliciting initiative and motivation
Respect for other professions
Acceptance and acknowledgment of others
Barriers to IPE facilitation
Staff not interested in IPE
Power relations among professionals
Hierarchy based on years of experience
Differences in perspectives between professions
Difficulties in maintaining IPE activities

## Discussion

We developed an IPE facilitation program based on an instructional design and tested its effectiveness. The results showed that the two-day program improved IPE facilitation skills and remained effective one year later. The qualitative analysis indicated that the program’s continued effectiveness was attributed to the knowledge and skills the participants learned in the program and applied in their workplaces. Additionally, the participants acquired skills in motivating and eliciting initiatives from staff members based on their respect for other professions and skills in IPE facilitation and conflict management.

The strength of this study is that the IPE facilitation program was developed based on the ARCS instructional design model. To date, no reports of other IPE facilitation programs developed on the basis of the ARCS model exist. There is strong relevance and reliability to the ARCS model because the participants analyzed real interprofessional collaboration issues in the context of their workplaces, thought about how to use IPE facilitation skills to address these issues, and practiced a simulation within the program [13,14].

Another strength of this study is that the IPE facilitation program’s learning effects continued for one year after the course. According to previous studies, educational programs developed based on the ARCS model were found to be effective at raising students’ attention during instruction, thereby developing their confidence in learning and improving their intrinsic and extrinsic motivation levels [17-19]. However, no other studies have achieved long-term results through programs based on the ARCS model. One of the learning strategies shown to help retain knowledge and skills over time is active learning [20,21]. Active learning encompasses a broad range of activities that require learners to construct, understand, and comprehend the knowledge derived from their educational experience while simultaneously engaging with it [20]. This program also included an active learning component, allowing participants to analyze the challenges of interprofessional collaboration in their own organizations and practice IPE facilitation in a simulated workshop. This may be one of the reasons why the learning effects of this program lasted for an extended period after the completion of the course.

The learning outcome of our IPE facilitation program can be evaluated at Level 3 using the Kirkpatrick model. Parsons et al. modified Kirkpatrick’s categories, an educational assessment model, for IPE outcomes: Level 1 is learner reaction, Level 2a is changes in attitudes/perceptions, Level 2b is the acquisition of knowledge/skills, Level 3 is behavioral change (the transfer of learning to the practice setting and changes in professional practice), Level 4a is organizational change, and Level 4b is the benefits to patients/clients [22]. The learning outcomes of our program can be rated as Level 3 because the participants’ IPE facilitation skills were retained for one year; furthermore, the open-ended answers to the questionnaire indicated that the participants continued to practice IPE facilitation in the workplace and that it was effective.

The limitations of the current study are, first, the small sample size and the limited types of professionals who participated, which restrict the generalizability of the study’s findings. Second, the two dropouts in the one-year survey may have been participants who were not practicing IPE facilitation. In this case, selection bias is possible in the IPFS scores after one year. Third, although the learning outcomes from this IPE facilitation program were considered to be Kirkpatrick’s Level 3, the participants’ performance in the workplace was self-assessed and not peer-assessed. Objective evaluations would have been possible through interviews with colleagues or work-based assessments.

## Conclusions

We developed a two-day IPE facilitation program based on the ARCS instructional design model and tested its effectiveness. The results showed that the participants’ IPE facilitation skills scores increased and were maintained one year later. The relevance of the knowledge and skills learned in the program, which were applied in the workplace, and the participants’ confidence in their ability to develop skills that would motivate other professionals and inspire them to develop initiatives were considered the keys to success.
